# The location of energetic compartments affects energetic communication in cardiomyocytes

**DOI:** 10.3389/fphys.2014.00376

**Published:** 2014-09-29

**Authors:** Rikke Birkedal, Martin Laasmaa, Marko Vendelin

**Affiliations:** Laboratory of Systems Biology, Institute of Cybernetics, Tallinn University of TechnologyTallinn, Estonia

**Keywords:** ADP, calcium, cardiomyocytes, creatine kinase, energetic compartments, mitochondria, oxidative phosphorylation, regulation

## Abstract

The heart relies on accurate regulation of mitochondrial energy supply to match energy demand. The main regulators are Ca^2+^ and feedback of ADP and P_i_. Regulation via feedback has intrigued for decades. First, the heart exhibits a remarkable metabolic stability. Second, diffusion of ADP and other molecules is restricted specifically in heart and red muscle, where a fast feedback is needed the most. To explain the regulation by feedback, compartmentalization must be taken into account. Experiments and theoretical approaches suggest that cardiomyocyte energetic compartmentalization is elaborate with barriers obstructing diffusion in the cytosol and at the level of the mitochondrial outer membrane (MOM). A recent study suggests the barriers are organized in a lattice with dimensions in agreement with those of intracellular structures. Here, we discuss the possible location of these barriers. The more plausible scenario includes a barrier at the level of MOM. Much research has focused on how the permeability of MOM itself is regulated, and the importance of the creatine kinase system to facilitate energetic communication. We hypothesize that at least part of the diffusion restriction at the MOM level is not by MOM itself, but due to the close physical association between the sarcoplasmic reticulum (SR) and mitochondria. This will explain why animals with a disabled creatine kinase system exhibit rather mild phenotype modifications. Mitochondria are hubs of energetics, but also ROS production and signaling. The close association between SR and mitochondria may form a diffusion barrier to ADP added outside a permeabilized cardiomyocyte. But *in vivo*, it is the structural basis for the mitochondrial-SR coupling that is crucial for the regulation of mitochondrial Ca^2+^-transients to regulate energetics, and for avoiding Ca^2+^-overload and irreversible opening of the mitochondrial permeability transition pore.

## Regulation of mitochondrial energy production in cardiomyocytes

The heart can never rest. During high workloads, skeletal muscle may develop an “energy debt,” which is paid back during rest. But the heart must avoid such an energy debt, as it cannot take a few minutes off to rest and restore its energy levels. Therefore, it is crucial to quickly and precisely regulate energy generation to match the energy consumption in time and space. The primary energy source in the heart is mitochondrial oxidative phosphorylation, which is mainly regulated by Ca^2+^ and ADP/P_i_-feedback.

Ca^2+^ exhibits “parallel regulation” of myofibrillar contraction and mitochondrial energy supply. Ca^2+^ enters the mitochondria through the Ca^2+^ uniporter, and is pumped out again mainly via the mitochondrial Na^+^/Ca^2+^-exchanger (Wei et al., 2011). This happens on a beat-to-beat basis. Mitochondrial Ca^2+^-transients have the same time to peak as cytosolic Ca^2+^-transients, but slower decay (Lu et al., 2013). Ca^2+^ in the mitochondrial matrix stimulates pyruvate, isocitrate and α-ketoglutarate dehydrogenase (McCormack et al., 1990), which reduce NAD to NADH in the citric acid cycle, as well as the F_1_F_0_-ATPase (Territo et al., 2000). Mitochondrial Ca^2+^-uptake increases with contraction frequency and adrenergic stimulation (Lu et al., 2013). Thus, as the Ca^2+^-transient increases to make cardiomyocytes contract faster and with greater force, so is the mitochondrial Ca^2+^-uptake enhanced to further stimulate mitochondrial energy generation.

ADP and P_i_, on the other hand, exhibit “feedback regulation” of mitochondrial energy supply. In contrast to Ca^2+^, which has a steep electrochemical gradient and is let into the cytosol and pumped out, the feedback regulation depends on the energetic circuit, where ATP diffuses from the mitochondria to the ATPases, and ADP and P_i_ diffuse from the ATPases to the mitochondria. The importance of feedback as a regulator of mitochondrial energy supply is intriguing, because the heart exhibits a remarkable metabolic stability: the ADP-concentration is unchanged even during large increases in workload and oxygen consumption (Katz et al., 1989; Balaban, 2002). P_i_-concentration changes the most, and some studies suggest P_i_ to be an important regulator, in particular in low to moderate workloads (Saks et al., 2000; Bose et al., 2003; Wu et al., 2008). Irrespectively, it is intriguing that specifically in cardiomyocytes and red muscle, where feedback regulation is needed the most, there seems to be barriers that obstruct diffusion significantly.

In working heart trabeculae, NADH fluorescence decreases and then partially recovers upon an increase in work (Brandes and Bers, 2002). This suggests that there are multiple regulators of mitochondrial energy production. As noted above, Ca^2+^ stimulates dehydrogenases to produce NADH. It also stimulates F_1_F_o_-ATPase. But overall, Ca^2+^-uptake by isolated mitochondria leads to an increase in NADH (Territo et al., 2001). NADH increases or decreases with P_i_ depending on the presence of ADP (Bose et al., 2003). ADP stimulates respiration rate, which decreases NADH fluorescence and increases flavoprotein fluorescence (Jepihhina et al., 2011). Cortassa and collaborators made an integrated model taking into account how excitation contraction coupling influences mitochondrial energetics (Cortassa et al., 2006). With this model, they were able to reproduce the experimental data of Brandes and Bers (2002) and analyze the regulatory mechanisms. Their quantitative analysis suggests that during work transitions energy supply is regulated initially by feedback, which decreases NADH. Subsequent parallel regulation by Ca^2+^ counterbalances this decrease, and NADH recovers (Cortassa et al., 2006). To explain how feedback can respond so quickly and be so important, despite overall metabolic stability, it is necessary to take into account energetic compartmentalization in cardiomyocytes.

It is well recognized that Ca^2+^ compartments exist in cardiomyocytes. Local Ca^2+^-events are visible with Ca^2+^-indicators (Wang et al., 2004). Due to the low Ca^2+^-affinity of the mitochondrial Ca^2+^-uniporter, mitochondrial Ca^2+^-uptake would not take place if it were not for the structural proximity between mitochondria and the sarcoplasmic reticulum (SR) (Franzini-Armstrong, 2007). There is, however, no direct coupling, and Ca^2+^ has to diffuse from the SR to the mitochondria (Franzini-Armstrong, 2007). This leads to an intra-mitochondrial Ca^2+^-gradient (Lu et al., 2013). The other major signaling molecule in the cell, cyclic AMP, is also confined to compartments. This is the only way to explain that stimulation of specific receptors using the same signaling cascade components (G_s_ proteins, cyclic AMP, and protein kinase A) leads to specific responses (Kritzer et al., 2012; Mika et al., 2012).

With these considerations in mind, it is not so surprising that cardiomyocytes also have energetic compartments with local concentrations of ADP, P_i_, and ATP. Actually, energetic compartmentalization of cardiac tissue was suggested already in 1970, where Gudbjarnason et al. showed that after induction of ischemia, cardiac contraction declines with the concentration of phosphocreatine (PCr), while overall ATP remains unchanged (Gudbjarnason et al., 1970). It is difficult to assess energetic compartments as there are no good fluorescent indicators for ADP, P_i_, and ATP (as for Ca^2+^). As an indirect measure, many studies of energetic compartmentalization have assessed and/or modeled mitochondrial function in permeabilized fibers and cardiomyocytes—traditionally during ADP- and ATP-titrations. Mitochondria in permeabilized cardiomyocytes are characterized by an apparent ADP-affinity that is much lower than that of isolated mitochondria. This is taken to indicate significant diffusion restriction between the medium outside the permeabilized cell and the adenine nucleotide translocase (ANT) in the mitochondrial inner membrane. Questions that are still being debated are: What causes the restriction of diffusion? And how does it affect energetic communication between ATPases and mitochondria via feedback?

The exact location of energetic compartments may differ from other molecules compartments, but the basic principles of compartment formation are the same. The concentration of a molecule in a given location depends on (1) the reaction rate and relative location of proteins involved in its production/release and consumption/uptake, (2) its diffusion speed, which in turn depends (among other things) on its concentration gradient, (3) its buffering by particulate and/or soluble proteins in the cell, and (4) the organization of physical barriers in the form of membrane structures, organelles, and macromolecular complexes, which may obstruct diffusion. If the sites of synthesis and consumption are close to each other, the molecule may be immediately consumed thus not being able to diffuse to other parts of the cell. Thus, the molecule concentration will be locally much higher compared to the bulk, and the enzymes processing the molecule are said to be coupled. This seems to be the mechanism regulating the compartmentalization of cyclic AMP (Kritzer et al., 2012; Mika et al., 2012). Any enzyme pair with common substrate/product can become coupled. One prerequisite is, however, that they are bound in each other's vicinity, so that the substrate/product is channeled between the enzymes within the unstirred layer immediately above the surface (Goldman and Katchalski, 1971; Arrio-Dupont et al., 1985; Fossel and Hoefeler, 1987; Arrio-Dupont, 1988). If the molecule is consumed further away from its generation site, it has enough time to diffuse. Then, its distribution in the cell depends more on diffusion speed, buffering, and physical structures forming diffusion barriers. Much of molecular motion in the cell occurs by diffusion (Kinsey et al., 2011). For example, diffusion of ROS is effective in the micrometer range, making it a feasible mechanism of communication between mitochondria (Aon et al., 2004).

## Energetic compartments in different sizes

Experimental data suggest there to be multiple energetic compartments scaling in size from coupled enzymes to the proposed intracellular energetic units (ICEUs) (Saks et al., 2001). Starting with the smallest, good examples of coupled enzyme pairs are those of creatine kinase (CK), which is bound near and coupled to various cellular ATPases such as myosin ATPase (Ventura-Clapier et al., 1987; Arrio-Dupont, 1988; Haagensen et al., 2008), the sarco-endoplasmic reticulum Ca^2+^-ATPase, i.e., SERCA (Minajeva et al., 1996), the Na^+^/K^+^ ATPase (Grosse et al., 1980), and the K_ATP_-channel (Crawford et al., 2002). Here, the ATPases hydrolyze ATP to ADP and P_i_, and CK uses PCr to regenerate ADP to ATP. In the mitochondria, the reaction goes the other way: ADP is phosphorylated to ATP, and the mitochondrial form of CK (Mi-CK) in the inter-membrane space uses creatine to regenerate ADP (Wallimann et al., 1992). Structural and model studies have confirmed that Mi-CK is bound near the ANT via its binding to cardiolipin in the inner mitochondrial membrane (Rojo et al., 1991; Schlattner et al., 2009; Karo et al., 2012), suggesting direct metabolite transfer between them (Vendelin et al., 2004b). The reaction may also go in the direction of ADP and PCr synthesis in the cytosol, where CK may be coupled to glycolytic enzymes (Kraft et al., 2000).

Larger compartments depend more on physical structures forming diffusion barriers. At the next size level, compartments are represented by organelles. In cardiac tissue, mitochondria are the organelles taking up the largest volume, 20–30%, whereas next in size the SR represents 4.5% of the cell volume (Decker et al., 1991). In mitochondria, the double membrane results in an inter-membrane as well as a matrix compartment. In cardiomyocytes, there is a significant barrier for ADP at the level of the mitochondrial outer membrane (MOM). This was first suggested based on the apparent ADP-affinity being much lower in permeabilized fibers than in isolated mitochondria (Saks et al., 1991, 1993; Kuznetsov et al., 1996). Indeed, adenine nucleotides pass the MOM through the voltage gated anion channel (VDAC) (Rostovtseva and Colombini, 1997), and the permeability of VDAC can be regulated by tubulin (Rostovtseva et al., 2008; Rostovtseva and Bezrukov, 2012).

In cardiomyocytes, even larger compartments have been proposed to exist. They were named ICEUs (Saks et al., 2001). Although their delimiters are still not identified, it has been suggested that t-tubules, organelles, and macromolecular complexes are organized in such a manner that ATPases are grouped with mitochondria. The existence of ICEUs was proposed on the basis of experiments suggesting the existence of cytoplasmic diffusion restrictions. Diffusion restriction by MOM can explain the much lower ADP-affinity in permeabilized cardiomyocytes. However, it cannot explain the fact that ADP and P_i_ generated inside permeabilized cardiomyocytes seem to be “channeled” to the mitochondria rather than out of the solution (Kummel, 1988; Seppet et al., 2001). For this, there has to be cytosolic diffusion restrictions as well. Modeling shows that these are localized rather than uniformly distributed in the cytoplasm (Vendelin et al., 2004a). A more elaborate 3D model shows a possible arrangement of the diffusion restrictions, which are at the level of MOM and as sheets between mitochondria—probably formed by the SR and cytoskeletal proteins (Ramay and Vendelin, 2009).

## Energetic compartments affect energetic communication—but how?

In the energetic circuit between ATPases and mitochondria, the main issue is how to efficiently transport ADP and P_i_ from ATPases to the mitochondria. This ensures an adequate phophorylation potential near ATPases and regulation of mitochondrial energy production. Overall, ADP ranges in the μM, whereas ATP, P_i_, PCr, and creatine range in the mM (Wallimann et al., 1992). P_i_-concentration varies the most with changes in work (Wu et al., 2008). However, due to its low concentration, even small changes in ADP have a large effect on the phosphorylation potential, which must be above a certain value for ATPases to obtain sufficient energy from ATP hydrolysis.

The physiological importance of coupled enzymes is quite obvious. The functional coupling between cytosolic CK and ATPases is beneficial when energy demand exceeds energy supply, and PCr is used to buffer the ADP/ATP-ratio and thus the phosphorylation potential. This situation has been dubbed “temporal energy buffering,” that is characterized by a net consumption of PCr to buffer ATP. In cardiomyocytes, the functional coupling between Mi-CK and ANT is beneficial, because oxidative phosphorylation is the main source of energy, which can be stored as PCr. The CK equilibrium constant favors ADP phosphorylation. Thus, locally high ATP concentrations or direct transfer of ATP from ANT is needed for Mi-CK to generate PCr. The same is true for cytoplasmic CK coupled to glycolytic enzymes.

On a slightly larger scale, the situation becomes more debatable. In addition to the temporal energy buffering, CK has been suggested to function as a spatial buffer. The spatial buffering occurs because the CK system forms an energy circuit with creatine and PCr, which runs in parallel with that of ADP and ATP. In the presence of spatial energy buffering there is no net consumption of high-energy phosphates, i.e., consumption matches generation. Thus, the CK system or “CK shuttle” facilitates energetic communication between ATPases and ATP-producing sites.

In the heart that depends on reliable regulation of mitochondrial energy, the CK system has been assumed by other investigators to be paramount. Indeed, it seems paradoxical that in oxidative muscles, that rely on energy generated by mitochondria, there is a significant barrier obstructing the feedback from ATPases at the MOM level (Kuznetsov et al., 1996; Ventura-Clapier et al., 1998). As an explanation, it has been proposed that the MOM permeability is regulated to ensure energetic communication via the CK system (Saks et al., 1994). While this proposal is appealing, the role of the CK system continues to be debated. As noted above, it has been shown that dimeric tubulin binds VDAC and can restrict its permeability (Rostovtseva and Bezrukov, 2012). But it has yet to be established whether such a restriction actually occurs *in vivo* and whether this restriction is regulated in the heart. If such regulation occurs, we would expect MOM to be more permeable in the absence of a functional CK system.

Experiments where the CK system was inhibited by feeding with beta-guanidinoproionic acid (a creatine analog) or knockout of one or more CK isoforms have shown varying effects on cardiac function. In general, the hearts seem to adapt to cope with basal workloads, but they fail under high workload conditions, fast work transitions and ischemia (Shoubridge et al., 1985; Mekhfi et al., 1990; Zweier et al., 1991; Neubauer et al., 1999; Kaasik et al., 2001; Crozatier et al., 2002; Spindler et al., 2004; Nahrendorf et al., 2005). In studies of knockout mice, genetic background has turned out to be important, and backcrossing seems to result in a milder phenotype (Lygate et al., 2009, 2012). It has been surprising that, so far, no compensatory changes have been found in heart of mice lacking guanidineacetate methyltransferase (GAMT; an enzyme in the creatine synthesis pathway), where the CK system is non-functional due to lack of creatine. These mice are smaller in size (Schmidt et al., 2004) but exhibit the same exercise capacity and tolerance to chronic myocardial infarction of their wild type littermates (Lygate et al., 2013). Furthermore, in a follow up study it was shown that intracellular compartmentalization as well as diffusion across MOM and mitochondrial organization were unchanged (Branovets et al., 2013). GAMT deficient mice accumulate guanidinoacetate, which is phosphorylated and may be used instead of creatine by CK in critical situations (Boehm et al., 1996; Kan et al., 2004). However, guanidinoacetate is not used by Mi-CK (Boehm et al., 1996), thus being unable to facilitate transport across MOM. Consequently, the cardiomyocytes of these mice do relatively well without a CK system, despite the fact that diffusion is restricted at the level of MOM to the same extent as in wildtype. This is in agreement with the idea that mitochondria are able to supply energy for SERCA-mediated Ca^2+^-uptake with the same efficiency as CK (Kaasik et al., 2001). However, this interpretation raises the following question: is the CK system needed to facilitate ADP/ATP transport across MOM in the heart? At present, we have no firm answer to this question, and further studies are needed.

It must be noted that spatial energy buffering by CK may not exclusively mean that CK facilitates energetic communication across MOM. Experiments with mice overexpressing M-CK showed that the higher M-CK activity and the associated higher CK flux significantly improves cardiac function after ischemia-reperfusion (Akki et al., 2012) and in failing hearts (Gupta et al., 2012). Indeed, the decrease in CK flux can be used as a predictor of heart failure (Bottomley et al., 2013). Taken together these data indicate that lack of CK does not worsen heart failure and that M-CK overexpression has therapeutic potential (Lygate and Neubauer, 2014). Whereas the rescue by M-CK overexpression seems to be at odds with the lack of changes in knockout models, differences in metabolism and energy transfer during energy starvation might provide an explanation. In the healthy heart, CK activity is moderate and a significant fraction is accounted for by Mi-CK. In glycolytic muscles, the total CK activity is higher than in oxidative muscle, mainly due to cytosolic M-CK (Ventura-Clapier et al., 1998). Both ischemic and failing hearts exhibit insufficient mitochondrial energy generation, relying on glycolytic energy supply to a large extent. This prompts us to ask whether hearts overexpressing M-CK are rescued because of a higher M-CK activity enabling a more efficient energy transfer between glycolytic enzymes and ATPases.

The importance of CK as a spatial energy buffer has also been studied using theoretical approaches. Some of these studies support the idea that CK-mediated enhancement of the ADP feedback is important (Wu and Beard, 2009). A model based on NMR data suggested that under normal conditions, energetic communication may occur via direct ADP/ATP transport as well as creatine/PCr transport. In contrast, at high workload, the CK system is bypassed (Vendelin et al., 2010). A recent study shows that energy transport via the CK system amounts to no more than 15% (Hettling and van Beek, 2011). In fact, temporal and spatial energy buffering by the CK system are inseparable (Meyer et al., 1984). This is in agreement with the idea that the mitochondrial response to a change in heart rate is faster in CK knockout mice (Gustafson and van Beek, 2002). In view of these data, we ask whether in the heart, spatial energy buffering is needed under physiological conditions, and for facilitated transport across MOM.

Model simulations of kinetic data suggest moderate diffusion restriction at the MOM level, but not necessarily by MOM itself (Ramay and Vendelin, 2009; Sepp et al., 2010). Thus, a significant part of the low apparent ADP-affinity in permeabilized cells is due to cytoplasmic diffusion restrictions forming ICEUs. ICEUs explain why ADP is channeled to mitochondria rather than out of the cell (Seppet et al., 2001), and why mitochondrial ATP is as efficient as CK in providing ATP to SR and myofilaments with ATP (Kaasik et al., 2001). Hypothetically, ICEUs confine mitochondria and ATPases in smaller compartments to reduce diffusion distances and ensure direct energetic communication. However, it remains an open question whether the ICEUs are “designed” in the sense that some structures are specifically organized in order to form ICEUs, or whether ICEUs are simply the consequence of cellular organization.

**Figure 1:**
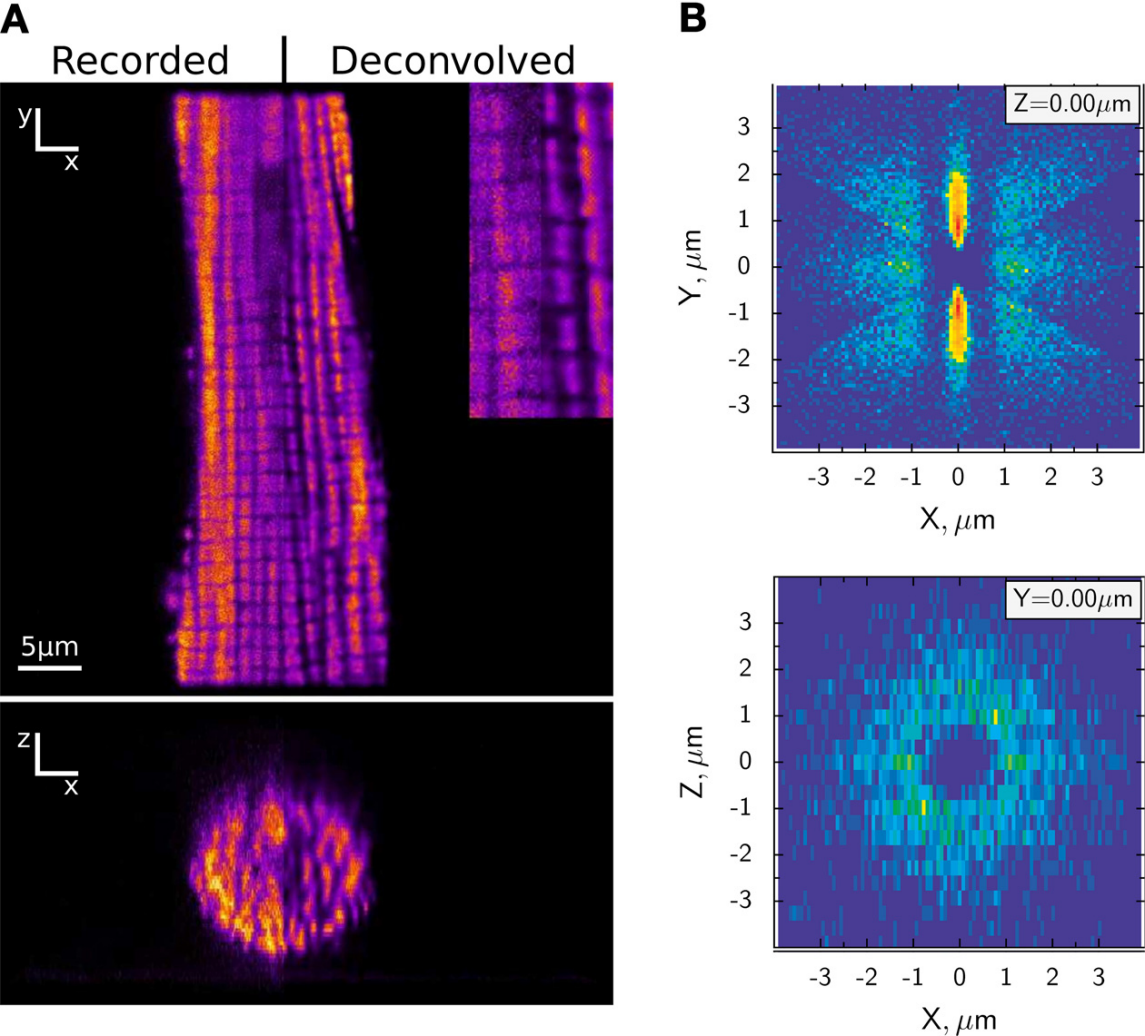
**Highly ordered arrangement of intermyofibrillar mitochondria in rat cardiomyocytes. (A)** Representative confocal image of MitoTracker Green labeled mitochondria are shown on the top (XY) and reconstructed cross-section (XZ) at the bottom. The original images are compared with deconvolved images after applying algorithms developed in Laasmaa et al. (2011). **(B)** Probability density of the closest mitochondrial centers in each sector of a rat cardiomyocyte, calculated as described in Birkedal et al. (2006). The density is shown in pseudo color with blue corresponding to regions where no neighboring mitochondria were found and red to the regions with high probability of finding the center of neighboring mitochondria. Note that mitochondria are arranged in a regular pattern (XY plane) with parallel rows separated by ~ 1.8 μm that can be found in any transversal direction relative to each other (XZ plane). For details of the analysis, see Birkedal et al. (2006).

Figure 1A shows the regular arrangement of mitochondria in a cardiomyocyte. Figure 1B displays the probability distribution of neighboring mitochondria around a central mitochondrion. Of note is the circular arrangement of mitochondria in the cross-section (Birkedal et al., 2006). Does this arrangement explain energetic coupling between mitochondria and ATPases? Rows of myofilaments are surrounded by rows of mitochondria that function as sinks for ADP and Pi. Oppositely, rows of mitochondria are also surrounded by rows of myofilaments that function as sinks for ATP. It seems natural that in a feedback system with energetic circuits, the majority of metabolites diffuse down their concentration gradients the shortest possible path rather than diffusing out of the permeabilized cell.

**Figure 2:**
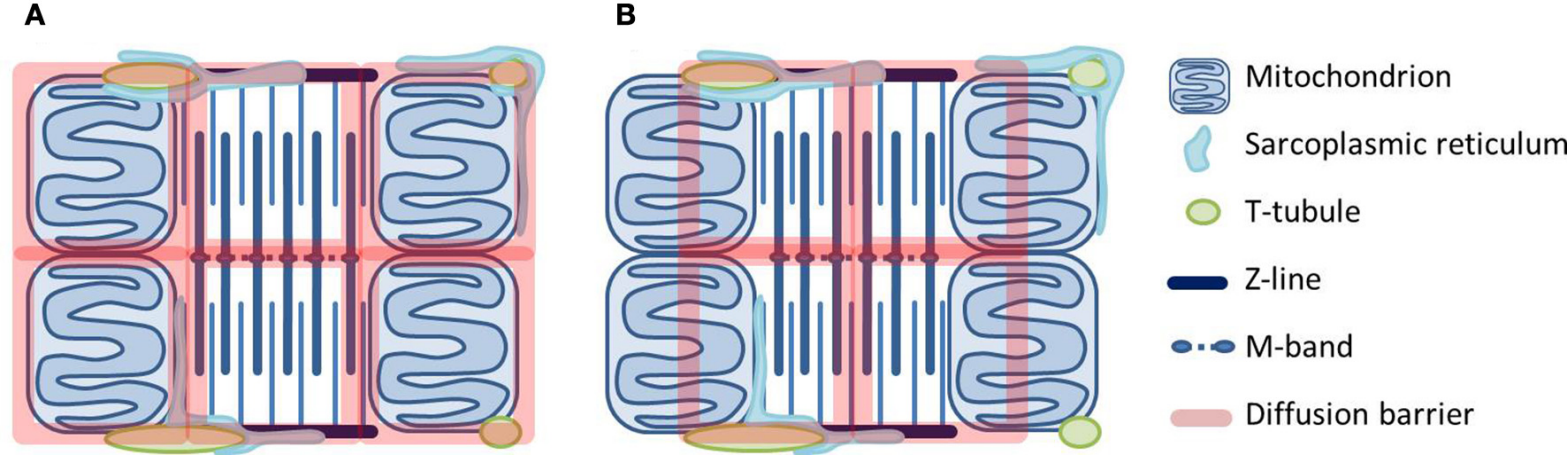
**Two scenarios for how diffusional barriers may be organized in cardiomyocytes**. The schematic drawings are scaled according to Birkedal et al. (2006), Hayashi et al. (2009) and show mitochondria, t-tubules and sarcoplasmic reticulum (SR) around a sarcomere. The diffusional barriers are drawn to scale according to Illaste et al. (2012) and superimposed. In **(A)** the barriers are in agreement with the cell structures, but seem to separate mitochondria and myosin ATPases. In **(B)** mitochondria are grouped together with ATPases, but this scenario is difficult to explain in structural terms.

In the quest for the location of cytosolic diffusion restrictions, we have studied intracellular diffusion using raster image correlation spectroscopy (RICS). We first showed that radial diffusion of fluorescently labeled ATP in cardiomyocytes is slower than transversal diffusion (Vendelin and Birkedal, 2008). A later study used two fluorescent molecules of different size (Illaste et al., 2012). Here, it was intriguing to find that in the cardiomyocyte compared to solution, diffusion of the large molecule was less restricted than diffusion of the small molecule. A stochastic model predicted that diffusion restrictions form a lattice with dimensions that are in agreement with the cardiomyocyte ultrastructure (Illaste et al., 2012). In Figure 2, we take a step further and draw this lattice superimposed on the cardiomyocytes ultrastructure.

Two possible scenarios are represented in Figure 2. In the first one (Figure 2A), the lattice is superimposed onto the mitochondrial membranes and the m-band and z-line of the sarcomere. In the second one (Figure 2B), half of a mitochondrion is grouped together with a fraction of a sarcomere. Considering the structures in the cell, the former seems more plausible: Transversally, diffusion barriers are formed by mitochondrial and SR membranes whereas longitudinal barriers are constituted by protein-dense regions in the myofilaments (z-lines and m-bands) and probably with some help from the junctional SR and t-tubules. However, the problem is that diffusion restriction at the level of MOM seems to separate rather than group together mitochondrial energy generation and ATPase energy consumption. An alternative explanation would be that diffusional restrictions at MOM are not due to the membrane itself but a result of its close association with the SR. Much of the work on energetic compartmentalization uses permeabilized cardiomyocytes, which are kept in a relaxed state. Although non-physiological, this is a useful preparation since it represents a simple situation. However, the importance of the SR is difficult to study in permeabilized cardiomyocytes, as SERCA is not active in this preparation (Sepp et al., 2014). As a membranous structure, the SR associated with the mitochondria will restrict diffusion from the medium to mitochondria in permeabilized cardiomyocytes. However, it also forms the structural basis for the energetic coupling between SERCA and mitochondria (Kaasik et al., 2001).

A fully developed cardiac CK system with a relatively high expression of mitochondrial as well as cytosolic CK isoforms is mainly found in adult mammals. The fact that mitochondrial CK seems absent in the hearts of lower vertebrates such as fish and frog can be explained by that they have a lower body temperature and cardiac performance, and depend more on glycolytic energy production (Ventura-Clapier et al., 1998; Birkedal and Gesser, 2006; Sokolova et al., 2009). Likewise, neonatal mammals with lower cardiac performance and higher reliance on glycolysis do not express Mi-CK (Hoerter et al., 1991, 1994; Tiivel et al., 2000). As the cardiomyocytes mature, they increase in diameter and develop from a relatively simple morphology to multiple parallel rows of myofibrils and mitochondria organized in a crystal-like pattern as we know it in adult cardiomyocytes (Vendelin et al., 2005; Birkedal et al., 2006; Anmann et al., 2014). In parallel, they develop t-tubules and a more elaborate SR (Sedarat et al., 2000; Dan et al., 2007) as their excitation contraction coupling changes to depend less on trans sarcolemmal Ca^2+^-transport and more on L-type Ca^2+^-influx to trigger Ca^2+^-release from the SR (Huang et al., 2008). During maturation the functional significance of cytosolic and Mi-CK increases (Hoerter et al., 1994). In light of the question whether CK facilitates transport across MOM, it is tempting to speculate that the increase in Mi-CK during development occurs simply because the cells transition from glycolytic to mitochondrial energy generation (Ostadal et al., 1999) and functional coupling of CK to an energy generation site is necessary for this reaction to happen in the direction of PCr synthesis. Probably, the concomitant decrease in apparent ADP-affinity is not for increasing energetic communication via the CK system, but due to the simultaneous development of the SR. The SR is less developed in lower vertebrates (Santer, 1985; Franzini-Armstrong and Boncompagni, 2011). In mammal heart, the SR develops significantly postnatally (Dan et al., 2007; Huang et al., 2008), and so does its association with mitochondria (Boncompagni et al., 2009). Thus, changes in SR-mitochondria interactions might provide an alternative explanation for the inter-species differences and developmental changes in the apparent ADP-affinity of permeabilized cardiomyocytes (Ventura-Clapier et al., 1998; Sokolova et al., 2009; Anmann et al., 2014). Additionally, the low apparent ADP-affinity is specific for oxidative muscles (Kuznetsov et al., 1996; Ventura-Clapier et al., 1998). The difference between muscles might be explained by distinct expression of tubulin that regulate VDAC gating (Varikmaa et al., 2014). Another explanation involves SR-mitochondria interaction that seems to be more prominent in muscles that are rich in mitochondria (Franzini-Armstrong, 2007).

The close association of SR and mitochondria is crucial for proper function and energetic regulation. By ensuring mitochondrial Ca^2+^-uptake, it allows for regulation of mitochondrial energy production by Ca^2+^ as well as ADP and P_i_. Moreover, it ensures sufficient energy supply to SERCA. Indeed, mitochondria are as efficient as CK in providing energy for SERCA function (Kaasik et al., 2001). Furthermore, as is the subject of this special issue, mitochondria are not only energetic but also redox hubs since they generate reactive oxygen species (ROS) with signaling potential. Mitochondrial Ca^2+^-uptake may take part in the regulation of ROS. As noted in the beginning, Ca^2+^ uptake by mitochondria increases NADH. Therefore, the Ca^2+^-uptake under physiological conditions may have similar effects as the addition of respiratory substrates: increase in NAD(P)H in turn used to reduce glutathion and thioredoxin pools, important antioxidant systems modulating mitochondrial ROS-emission (Garcia et al., 2010; Stanley et al., 2011; Aon et al., 2012). Compromised energy provision affects SERCA activity and Ca^2+^-re-uptake. This may lead to an increase in mitochondrial Ca^2+^, which is known to be an important trigger of the permeability transition pore (mPTP) (Bernardi, 1999). mPTP opening is to some extent reversible. But high enough Ca^2+^ and stimulation by other factors leads to irreversible opening, and eventually to apoptosis and/or necrosis (Di Lisa et al., 2011). Functional coupling between SR and mitochondria will ensure energy supply to SERCA thus preventing excessive mitochondrial Ca^2+^-uptake. In a situation of limited energy supply, transport of ADP and P_i_ from SERCA to the mitochondria may increase the mitochondrial Ca^2+^-uptake capacity before mPTP opening is triggered (Wei et al., 2012; Sokolova et al., 2013).

## Concluding remarks

Feedback regulation of energetics depends on the location of diffusion barriers. We suggest an alternative explanation for the diffusion restriction at MOM level, namely that it is due to a close association of mitochondria and SR that ensures SERCA energy supply as well as mitochondrial regulation by Ca^2+^. It has been suggested that in cardiomyocytes, the permeability of MOM itself is regulated by tubulin. Indeed, “closing the gates” to the mitochondria to enhance energetic communication by the CK system seems like a good explanation, if it is more efficient. There are, however, some studies suggesting that a significant fraction of the energetic communication can occur as direct transport of ATP, ADP, and Pi. Also, cardiac function is not severely compromised by the lack of a functional CK system although diffusion restriction at the level of MOM is unchanged.

Whether the compartmentalization of energy units control the feedback exerted by the CK system or merely exists as part of a structural organization to optimize energy transfer between mitochondria and ATPases via ADP, P_i_, and Ca^2+^, remain open questions.

### Conflict of interest statement

The authors declare that the research was conducted in the absence of any commercial or financial relationships that could be construed as a potential conflict of interest.
